# Inhibition of ERK1/2 Worsens Intestinal Ischemia/Reperfusion Injury

**DOI:** 10.1371/journal.pone.0076790

**Published:** 2013-09-20

**Authors:** Kechen Ban, Zhanglong Peng, Rosemary A. Kozar

**Affiliations:** Department of Surgery, University of Texas Health Science Center at Houston, Houston, Texas, United States of America; Hungarian Academy of Sciences, Hungary

## Abstract

**Background:**

The role of extracellular signal-regulated protein kinase (ERK) in intestinal ischemia/reperfusion (I/R) injury has not been well investigated. The aim of the current study was to examine the effect of inhibition of the ERK pathway in an in vitro and in vivo model of intestinal I/R injury.

**Methods:**

ERK1/2 activity was inhibited using the specific inhibitor, U0126, in intestinal epithelial cells under hypoxia/reoxygenation conditions and in mice subjected to 1 hour of intestinal ischemia followed by 6 hours reperfusion. In vitro, cell proliferation was assessed by MTT (3-(4,5-dimethylthiazol-2-yl)-2,5-diphenyl tetrazolium bromide) assay, apoptosis by DNA fragmentation, and migration using an in vitro model of intestinal wound healing. Cells were also transfected with a p70S6K plasmid and the effects of overexpression similarly analyzed. In vivo, the effects of U0126 on intestinal cell proliferation and apoptosis, intestinal permeability, lung and intestinal neutrophil infiltration and injury, and plasma cytokine levels were measured. Survival was also assessed after U0126. Activity of p70S6 kinase (p70S6K) was measured by Western blot.

**Results:**

In vitro, inhibition of ERK1/2 by U0126 significantly decreased cell proliferation and migration but enhanced cell apoptosis. Overexpression of p70S6K promoted cell proliferation and decreased cell apoptosis. In vivo, U0126 significantly increased cell apoptosis and decreased cell proliferation in the intestine, increased intestinal permeability, intestinal and lung neutrophil infiltration, and injury, as well as systemic pro-inflammatory cytokines, TNF-α, IL-6 and IL-1β. Mortality was also significantly increased by U0126. Inhibition of ERK1/2 by U0126 also abolished activity of p70S6K both in vitro and in vivo models.

**Conclusion:**

Pharmacologic inhibition of ERK1/2 by U0126 worsens intestinal IR injury. The detrimental effects are mediated, at least in part, by inhibition of p70S6K, the major effector of mammalian target of rapamycin pathway.

## Introduction

Intestinal ischemia/reperfusion (I/R) occurs in a wide range of clinical scenarios, including small bowel transplantation, midgut volvulus, major trauma, thrombosis of the superior mesenteric artery and vein, hemorrhage and cardiopulmonary bypass. Intestinal I/R leads to the release of cytotoxic substances from the ischemic tissues and the accumulation of inflammatory mediators in the intestine. This results in mucosal injury, hyperpermeability, and inflammation. The release of proinflammatory factors and bacteria-derived endotoxins from the injured intestine may also instigate a systemic inflammatory response and lead to multiple organ dysfunction syndrome [1-3]. Therefore, intestinal I/R is associated with considerable morbidity and mortality [4].

The extracellular signal-regulated protein kinases (ERK) 1 and 2 (ERK1/2) signaling pathway is a cascade consisting of at least three families of protein kinases, including Raf (MAPKKK or MEKK), MAPKKs (MEK 1 and MEK 2), and MAPK (ERK 1 and ERK 2 or p42/p44 MAPKs). Each molecule acquires protein kinase activity after phosphorylation, and is subsequently able to phosphorylate and then activate the next member of the signaling cascade. The ERK pathway not only regulates a wide range of cell behaviors, such as, growth, proliferation, migration, differentiation, apoptosis and autophagy, but also mediates inflammatory responses [5,6]. The ERK pathway can be activated by a variety of extracellular stimuli such as growth factors, cytokines, mitogens, hormones, and oxidative or heat stress [7].

ERK1/2 has been implicated in tissue injury induced by I/R, but results have been inconsistent. Zhu et al. demonstrated that activation of ERK1/2 mediated neuroprotection of dexmedetomidine, a potent and highly selective α_2_-adrenoceptor agonist, in transient cerebral I/R [8]. Similarly, Wang et al. showed cardioprotection by pioglitazone, a potent peroxisome proliferator-activated receptor gamma (PPARγ) agonist that also activated ERK1/2 [9]. However, Lu et al. demonstrated that inhibition of ERK1/2 provided neuroprotection in spinal cord I/R [10]. Similarly, remifentanil, a potent synthetic µ-receptor agonist, improved functional outcome through, in part, attenuation of ERK1/2 activity in a transient cerebral I/R model [11]. There is limited evidence on the role of ERK pathway in the intestine, an organ at high risk for l I/R-induced injury [12]. Therefore, in the present study, we explored the effect of inhibition of ERK1/2, the central component of ERK pathway, in an in vitro and in vivo model of on intestinal I/R, on intestinal and lung injury.

## Materials and Methods

### Cell culture

The intestinal epithelial IEC-6 cell line which was derived from adult rat intestinal crypts was purchased from the American Type Culture Collection. Cells were maintained in Dulbecco’s modified Eagle’s medium supplemented with 10% fetal bovine serum (FBS), 2 mM l-glutamine, 1% penicillin and 1% streptomycin in the incubator with a humidified atmosphere of 5% CO_2_/95% air at 37°C. Cells were used between passages 17 and 25.

### Cell transfection with p70S6K plasmid

Cells were transfected with empty vector pRK7 plasmid, p70S6K plasmid (wild-type p70S6K expressing vector pRK7-HA-S6K1-WT) [13] using the Lipofectamine 2000 transfection reagent (Invitrogen) as described previously [14].

### In vitro model of I/R

A well-established hypoxia/reoxygenation (H/R) model was employed using IEC-6 cells to mimic I/R in vivo [14]. After incubation with serum-free medium in a hypoxic chamber with 0.5% O_2_/5% CO_2_/94.5% N_2_ for indicated periods of time, cells were reoxygenated by placing cells into in an incubator under 37°C/ 5% CO_2_ conditions with 10% serum (normoxic conditions) for indicated periods of time. To inhibit ERK1/2 phosphorylation/activity, cells were treated with 10 µM of U0126 (Sigma) [15,16], a specific inhibitor of ERK1/2, or vehicle (dimethyl sulfoxide) at the onset of normoxia for 1hour followed by 10% FBS and cultured under normoxic conditions for indicated periods of time.

### Assessment of cell growth in vitro

MTT (3-(4,5-dimethylthiazol-2-yl)-2,5-diphenyl tetrazolium bromide) assay was employed for cell growth assessment [14]. Briefly, 5 Χ 10^3^ cells were added into each well in a 96-well plate. After 24 hours, cells were cultured in medium without FBS under hypoxic conditions for 12 hours in a hypoxic chamber, and then 10 µM of U0126 or vehicle was added into cells. After 1 hour, 10% FBS was supplemented to medium. Cells were transferred to an incubator under normoxic conditions for 3 days. Cells were then incubated with MTT (Sigma) (20 µl/well, 5 mg/ml in phosphate buffered saline [PBS]) for 3 hours. After removal of medium, dimethyl sulfoxide (200 µl/well) was added to wells. A microplate reader was used to measure the absorbance of dissolution sample at 570 nm and subtracted background at 670 nm.

### Evaluation of DNA fragmentation in vitro

A Cell Death Detection ELISA (enzyme-linked immunosorbent assay) kit (Roche) was used to measure the DNA fragmentation in cells [14]. Cells were seeded in 96-well plates (1 Χ 10^4^ cells/well). After 24 hours, fresh FBS-free medium containing U0126 (10 µM) or vehicle was added. Cells were cultured under hypoxic conditions for 24 hours followed by incubation in normoxic conditions for 6 hours. To measure the DNA fragmentation, the medium was discarded, and the attached cells were washed twice with PBS and lysed with lysis buffer (200 µl /well). Cell lysates were collected into 1.5ml-tubes and centrifuge at 200 Χ g for 10 minutes. A 100-µl supernatant of the cell lysate per well was taken and added into the ELISA plate and measured the immunocomplex at 405/490 nm in a microplate reader. The value was normalized by cell numbers in corresponding wells in parallel experiments. Cells grown in basal conditions served as untreated control.

### Measurement of cell migration in vitro

A H/R model of in vitro intestinal wound healing was employed to mimic I/R in vivo [14]. Each well was seeded with 1X10^5^ cells in a 12-well plate for 24 hours. Cells were then cultured with FBS-free medium under hypoxic conditions for additional 12 hours. The confluent cell monolayer was scraped with a pipette tip across the diameter of the well. After removal of cell debris by washing with PBS two times, cells were then treated with U0126 (10 µM) or vehicle for 1 hour followed by adding 10% FBS. Cells were cultured for another 24 hours under normoxic conditions. The wound area at the beginning of wounding and at the end of treatment was photographed respectively under an inverted phase-contrast microscope with an attached camera. The wound area was measured using OPTIMAS 6.1 image analysis software (Optimas Corp.). The recovered surface area was calculated by subtracting the wound area at the end of treatment from the corresponding original wound area.

### Ethics statement

All animal protocols were approved by the University of Texas, Houston Medical School Animal Welfare Committee.

### Rodent model of intestinal I/R

Male, 8-10 weeks old C57BL/6J mice were randomly assigned into 4 groups (n=5/group): sham, sham-U0126, IR, IR-U0126. Mice were fasted with free access to water overnight before laparotomy. U0126 (1 mg/Kg, IP) [17] was given 30 minutes prior to surgery in sham-U0126 and IR-U0126 groups. Mice in sham and IR groups received equal volume of vehicle. Intestinal I/R was performed in IR and IR-U0126 groups as we have described [14]. Briefly, under general anesthesia with isoflurane-inhaled anesthetic, a midline laparotomy was made and the superior mesenteric artery occluded with a microclip to interrupt the blood flow to the intestine for one hour then release to re-establish blood flow. Sham and sham-U0126 groups underwent an identical procedure without I/R. Animals were sacrificed after six hours of reperfusion. Plasma, intestinal and lung tissues were harvested for analysis. For survival experiments, mice were given with buprenorphine (0.1 mg/kg, intraperitoneally, every 12 h) after incision was closed to minimize the pain, distress or discomfort. Animals were observed at least 3 times per day for 7 days. Any animal that was unable to right itself within 20 seconds when placed on its side was euthanized by CO_2_ asphyxiation immediately and recorded as dead.

### Examination of morphological injury in the intestine and lung

Intestinal and lung tissues were fixed in buffered formaldehyde solution (10% in PBS) for 24 hours and embedded in paraffin. The tissue was then sectioned, deparaffinized, stained with hematoxylin and eosin (HE). To evaluate histopathologic changes in the intestine, Park/Chiu scoring system was used in a blinded manner using a light microscope on a 0 to 5 scale, where 5 is the most severely injured [14,18]. Histological evaluation of lung injury using the system described by Feinman et al. [19], where 0 is normal and 5 is the most severe injury.

### Evaluation of myeloperoxidase (MPO) activity in the intestine and lung

A commercial Myeloperoxidase (MPO) Colorimetric Activity Assay Kit (BioVision Research Products) was employed to quantitatively measure the MPO activity in the intestinal and lung tissue. The MPO activity reflects the extent of neutrophil infiltration into the tissue. The MPO value was normalized to milligrams of protein determined by Bio-Rad Protein Assay (Bio-Rad Laboratories).

### Detection of serum cytokines

The relative level of cytokines TNF-α, IL-6 and IL-1β in plasma was determined by enzyme-linked immunosorbent assay with a Mouse Cytokine ELISA Strip I for Profiling 8 Cytokines kit (Signosis) according to manufacturers’ instruction.

### Assessment of intestinal permeability

The ex-vivo isolated everted ileum sac method [20] was used to evaluate the intestinal permeability as described previously [14].

### Analysis for protein expression in cells and intestines

Intestinal tissues and IEC-6 cells were quickly lysed with radioimmunoprecipitation assay buffer in the presence of protease inhibitors. Western blot analysis was performed as previously described [14]. Antibodies against p44/42 MAPK (Erk1/2), phospho-p44/42 MAPK (Erk1/2), p70S6K, phospho-p70S6K (p-p70S6K) and β-actin (all were from Cell Signaling Technology) were used.

### Evaluation of apoptotic cells in intestines

Cell apoptosis in the intestinal tissue was evaluated using an In Situ Cell Death Detection Kit (Roche) as described previously [14].

### Assessment of cell proliferation in intestines

Ki-67 is a nuclear protein that is expressed in proliferating cells. It has been used as a marker for cell proliferation. Immunohistochemistry for Ki-67 (anti-Ki-67 antibody, 1:100) (Merck Millipore) was performed using the Dako-Cytomation EnVision + System-HRP (AEC) kit (Dako) in paraffin-embedded intestinal sections as described previously [14]. Ki-67 proliferation index was defined as percent Ki-67 nuclear positive cells in a total 500 positive and negative cells in the area with highest Ki-67 labeling in each section.

### Statistical analysis

Data is presented as mean ± SEM and were compared by one way analysis of variance with the nonparametric Mann-Whitney test used to determine differences between groups. The Mantel–Haenzel log rank test was used to compare survival rates. A p value less than 0.05 was considered statistically significant.

## Results

### U0126 inhibited ERK1/2 activity in vitro and in vivo

**Figure 1:**
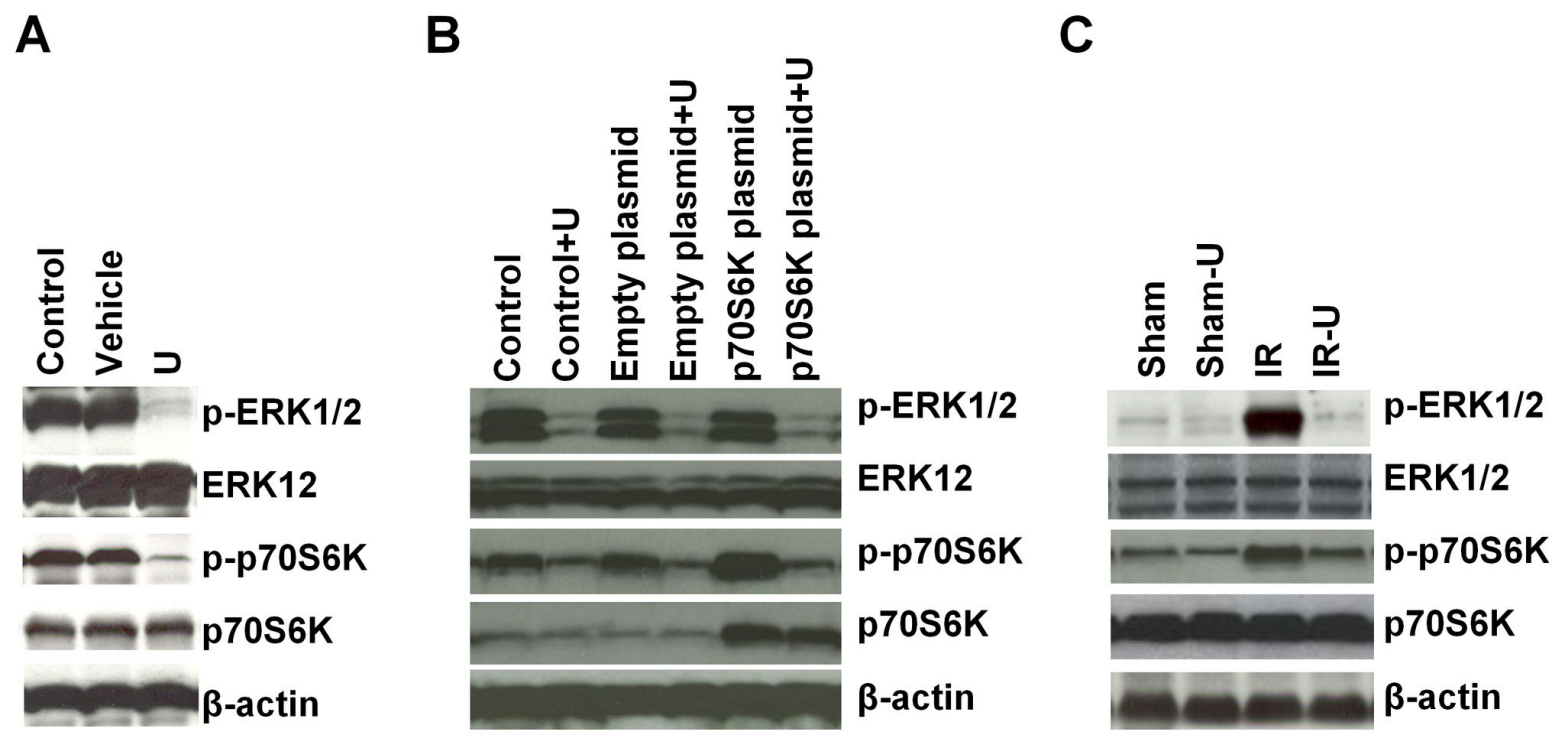
Activities of ERK1/2 and p70S6K were inhibited by U0126 in vitro and in vivo. A. IEC-6 cells were cultured in medium without FBS for 12 hours under hypoxic conditions followed treatment with U0126 or vehicle for 1 hour and then stimulated with 10% FBS under normoxic conditions for 20 minutes (n=3). Protein expression in cells was determined by Western blot analysis. B. IEC-6 cells were transfected with/without empty plasmid or p70S6K plasmid for 24 hours and cultured in medium without FBS for 12 hours under hypoxic conditions followed treatment with U0126 or vehicle for 1 hour and then stimulated with 10% FBS under normoxic conditions for 20 minutes (n=3). Protein expression was determined by Western blot analysis. C. Mice were pretreated with U0126 or vehicle and then subjected to one hour ischemia followed by 6 hours reperfusion in the intestine (n=5). Protein expression in the intestine was determined by Western blot analysis. U=U0126, Sham-U=Sham-U0126, IR=I/R, IR-U=I/R-U0126.

ERK1/2 is activated by phosphorylation. To test the efficacy of U0126 inhibition of ERK1/2 in intestinal cells under H/R conditions and after I/R in the intestines, we detected protein levels of phosphorylated ERK1/2 by Western blot. U0126 virtually abolished the phosphorylation of ERK1/2 in IEC-6 cells under H/R conditions (Figure 1A and 1B). In vivo, ERK1/2 was activated after I/R but activation was significantly attenuated by U0126 (Figure 1C).

### Inhibition of ERK1/2 reduced intestinal cell proliferation and migration and promoted apoptosis in vitro

**Figure 2:**
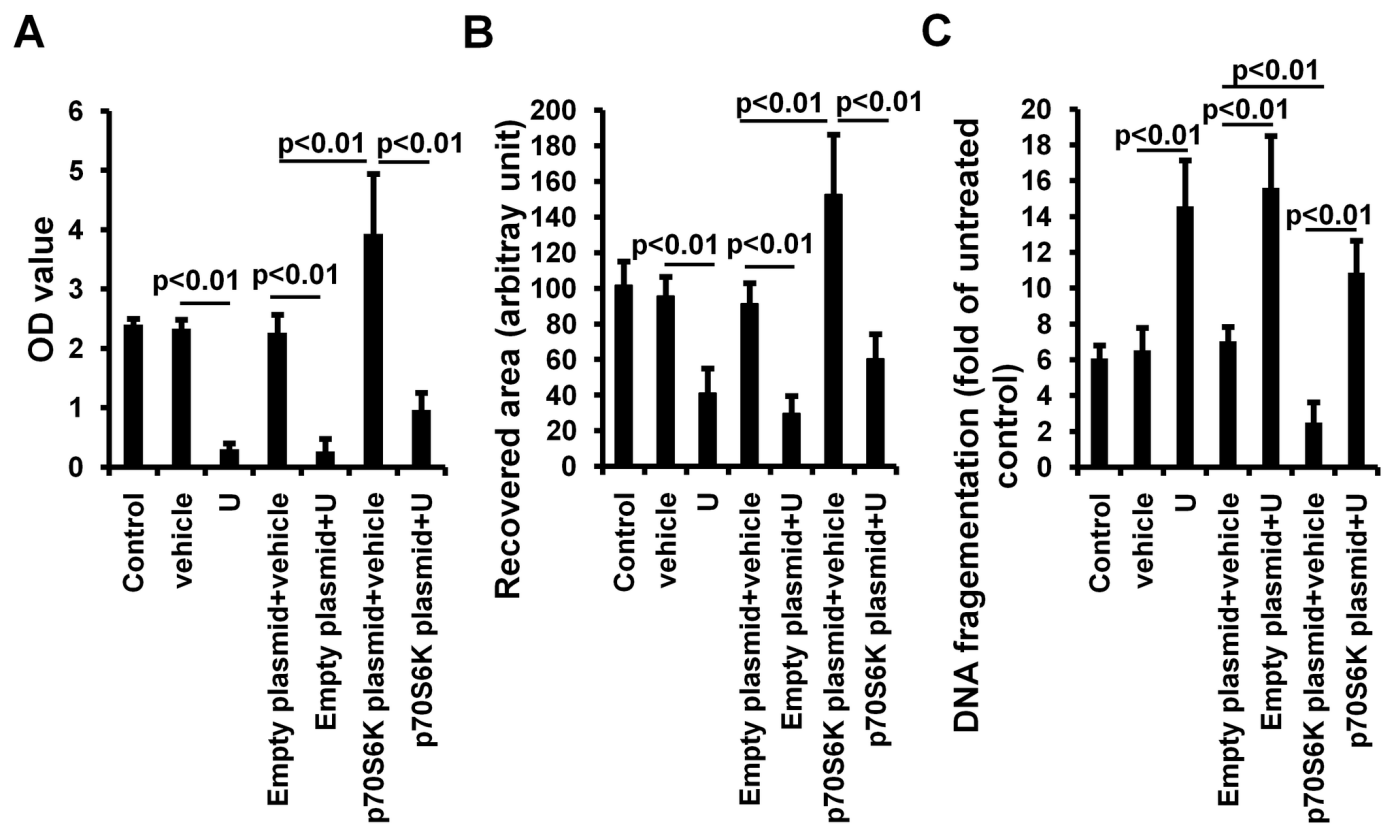
Inhibition of ERK1/2 and p70S6K by U0126 decreased cell proliferation and migration, and promoted cell apoptosis in vitro (n=3). A. After transfection with/without empty plasmid or p70S6K plasmid for 12 hours, IEC-6 cells were cultured in medium without FBS for 12 hours under hypoxic conditions followed by treatment with U0126 or vehicle for 1 hour and then stimulated with 10% FBS under normoxic conditions for 3 days. Cell proliferation was measured by MTT assay. B. After transfection with/without empty plasmid or p70S6K plasmid for 12 hours, IEC-6 cells were cultured in medium without FBS in a 12-well plate for 12 hours under hypoxic conditions. The confluent cell monolayer was then scraped with a 1-ml pipette tip. Cells were then treated with U0126 or vehicle for 1 hour followed by adding 10% FBS under normoxic conditions for 24 hours. The recovery area was measured. C. After transfection with/without empty plasmid or p70S6K plasmid for 12 hours, IEC-6 cells were cultured in FBS-free medium with U0126 or vehicle under hypoxic conditions for 24 hours followed by incubation in normoxic conditions for 6 hours. Cell apoptosis was determined by evaluation of DNA fragmentation. U=U0126.

Under H/R conditions, there was no difference between control and vehicle treated groups in cell proliferation (control vs. vehicle: 2.40±0.10 vs. 2.33±0.15. P>0.05), migration (control vs. vehicle: 101.67±13.31 vs. 95.67±10.69. P>0.05) or apoptosis (control vs. vehicle: 6.07±0.73 vs. 6.53±1.27. P>0.05) (Figure 2). U0126 significantly decreased cell proliferation (vehicle vs. U0126: 2.33±0.15 vs. 0.30±0.10, p<0.01) (Figure 2A), inhibited cell migration to the wound area (vehicle vs. U0126: 95.67±10.69 vs. 41.0±14.18, p<0.01) (Figure 2B) and increased cell apoptosis (vehicle vs. U0126: 6.53±1.27 vs. 14.57±2.57, p<0.01) (Figure 2C), suggesting a crucial role of ERK1/2 in regulation in cell proliferation, migration and apoptosis.

### Inhibition of ERK1/2 promoted cell apoptosis and decreased cell proliferation in vivo

**Figure 3:**
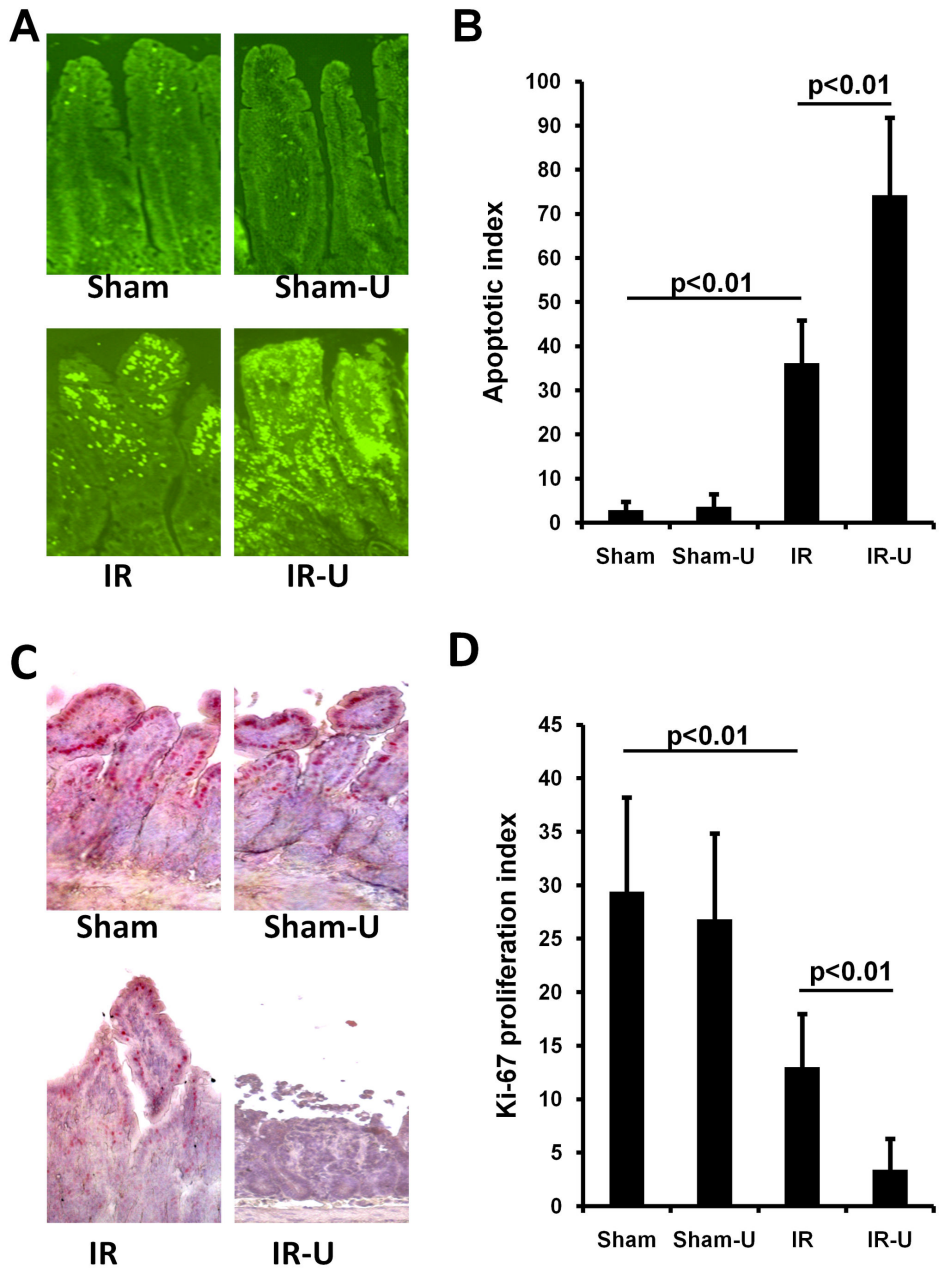
Inhibition of ERK1/2 by U0126 increased intestinal cell apoptosis and decreased cell proliferation in vivo. Mice were pretreated with U0126 or vehicle and then subjected to one hour ischemia followed by 6 hours reperfusion in the intestine (n=5). A. Representative images of apoptotic cells in the intestinal tissue detected using an In Situ Cell Death Detection Kit. B. Quantitative measurement of apoptotic cells in the intestinal tissue. C. Representative images of immunohistochemical staining for Ki-67 in the intestinal tissue. Immunohistochemical staining was performed using the Dako-Cytomation EnVision + System-HRP (AEC) kit. C. Quantitative measurement of immunohistochemical staining for Ki-67 in the intestinal tissue. Sham-U=Sham-U0126, IR=I/R, IR-U=I/R-U0126.

In sham groups, treatment of U0126 did not affect intestinal cell apoptosis (Sham vs. Sham-U0126: 2.80±1.92 vs. 3.60±2.88, p>0.05) or proliferation (Ki-67 proliferation index) (Sham vs. Sham-U0126: 29.40±8.82 vs. 26.80±8.04, p>0.05). However, after intestinal I/R, U0126 significantly enhanced intestinal cell apoptosis (IR vs. IR-U0126: 36.20±9.68 vs. 74.20±17.60, p<0.01) and reduced cell proliferation (IR vs. IR-U0126: 13.0±4.95 vs. 3.40±2.88, p<0.01) (Figure 3). These data demonstrate that inhibition of ERK1/2 also promotes cell apoptosis and suppresses cell proliferation after intestinal I/R.

### Inhibition of ERK1/2 increased intestinal inflammation, permeability and injury in vivo

**Figure 4:**
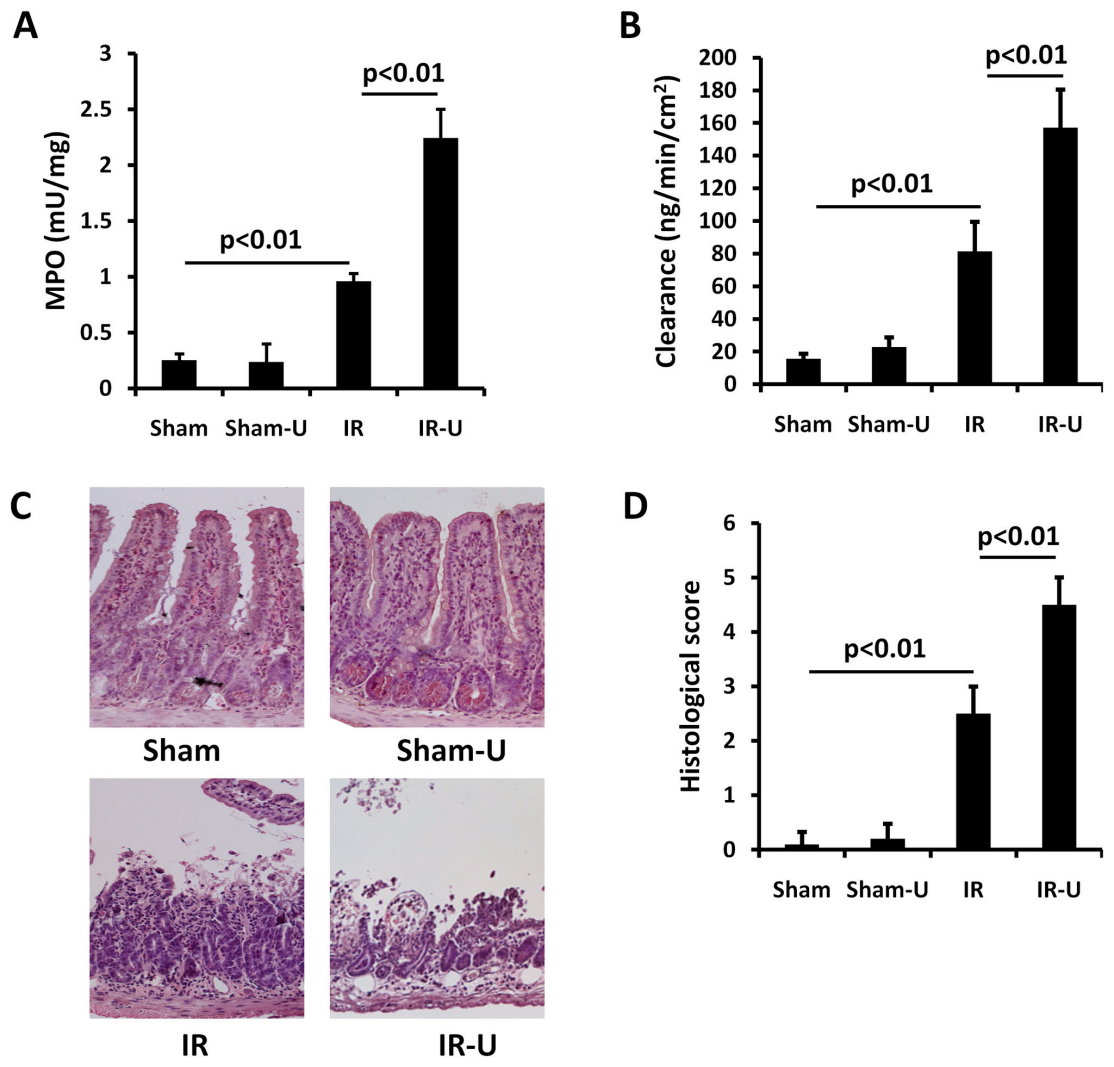
Inhibition of ERK1/2 by U0126 increased intestinal inflammation, permeability and injury in vivo. Mice were pretreated with U0126 or vehicle and then subjected to one hour ischemia followed by 6 hours reperfusion in the intestine (n=5). The intestinal tissue was examined. A. MPO activity by a commercial Kit. B. Permeability by the ex-vivo isolated everted ileum sac method. C. Representative images by HE staining. D. Quantitative tissue damage by Park/Chiu scoring system. Sham-U=Sham-U0126, IR=I/R, IR-U=I/R-U0126.

MPO activity, an index of neutrophil infiltration, was similar in the sham and sham-U0126 groups (sham vs. sham-U0126: 0.25±0.06 vs. 0.24±0.16. P>0.05). However, MPO activity was markedly increased in the intestine after I/R (sham vs. IR: 0.25±0.06 vs. 0.96±0.07. p<0.01) and was further increased by U0126 treatment (IR vs. IR-U0126: 0.96±0.07 vs. 2.24±0.26. p<0.01) (Figure 4A).

Permeability was similar in the intestine between sham and sham-U0126 groups (sham vs. sham-U0126: 17.64±1.99 vs. 22.86±5.90P>0.05), but was increased after I/R (sham vs. IR: 17.64±1.99 vs. 81.30±18.16, p<0.01) and U0126 treatment exacerbated the intestinal permeability (IR vs. IR-U0126: 81.30±18.16 vs. 157.08±23.30. p<0.01) (Figure 4B).

Similarly, there was no difference between sham and sham-U0126 in mucosal injury (sham vs. sham-U0126: 0.10±0.22 vs. 0.20±0.27. P>0.05), but I/R induced severe mucosal injury in the intestine (sham vs. IR: 0.10±0.22 vs. 2.50±0.50. p<0.01), and treatment with U0126 further worsened intestinal injury (IR vs. IR-U0126: 2.50±0.50 vs. 4.50±0.50. p<0.01) (Figure 4C and 4D).

### Inhibition of intestinal ERK1/2 increased systemic proinflammatory cytokines in vivo

**Figure 5:**
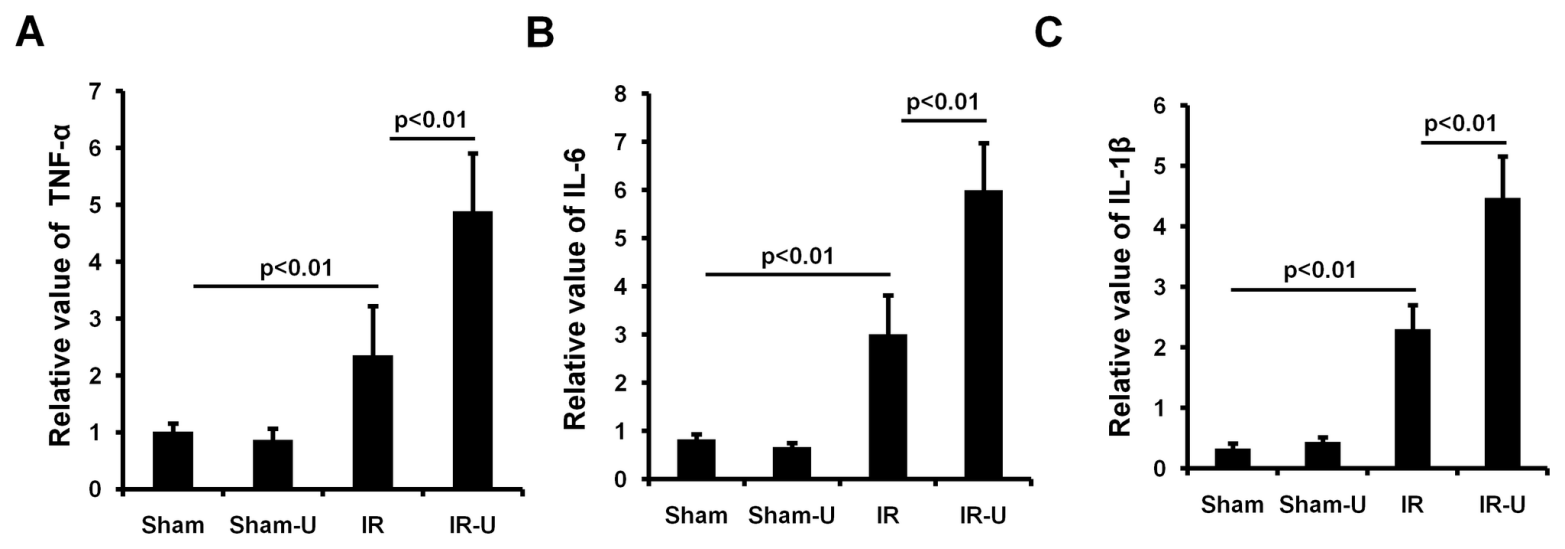
Inhibition of ERK1/2 by U0126 increased the levels of proinflammatory cytokines in plasma. Mice were pretreated with U0126 or vehicle and then subjected to one hour ischemia followed by 6 hours reperfusion in the intestine (n=5). The relatively levels of cytokines in plasma was determined using a commercial kit. A. The relatively level of cytokines TNF-α in plasma. B. The relatively level of cytokines IL-6 in plasma. C. The relatively level of cytokines IL-1β in plasma. Sham-U=Sham-U0126, IR=I/R, IR-U=I/R-U0126.

Consistent with previous reports [14,21], systemic pro-inflammatory mediator production was increased after intestinal I/R. TNF-α (sham vs. IR: 1.01±0.14 vs. 2.36±0.87. p<0.01), IL-6 (sham vs. IR: 0.83±0.11 vs. 3.01±0.81. p<0.01) and IL-1β (sham vs. IR: 0.33±0.08 vs. 2.31±0.39. p<0.01) were all increased after I/R(Figure 5) and further increased by U0126 (TNF-α :IR-U0126 vs. IR 4.87±1.02 vs. 2.36±0.87. p<0.01; IL-6:IR-U0126 vs. IR 5.99±0.98 vs. 3.01±0.81. p<0.01; and IL-1β: IR-U0126 vs. IR 4.47±0.69 vs. 2.31±0.39. p<0.01). There was no difference between sham groups in the levels of TNF-α (sham vs. sham-U0126: 1.01±0.15 vs. 0.87±0.20. P>0.05), IL-6 (sham vs. sham-U0126: 0.83±0.11 vs. 0.67±0.09. p>0.05) and IL-1β (sham vs. sham-U0126: 0.33±0.08 vs. 0.44±0.08. p>0.05) in plasma (Figure 5).

### Inhibition of ERK1/2 increased lung inflammation and injury in vivo

**Figure 6:**
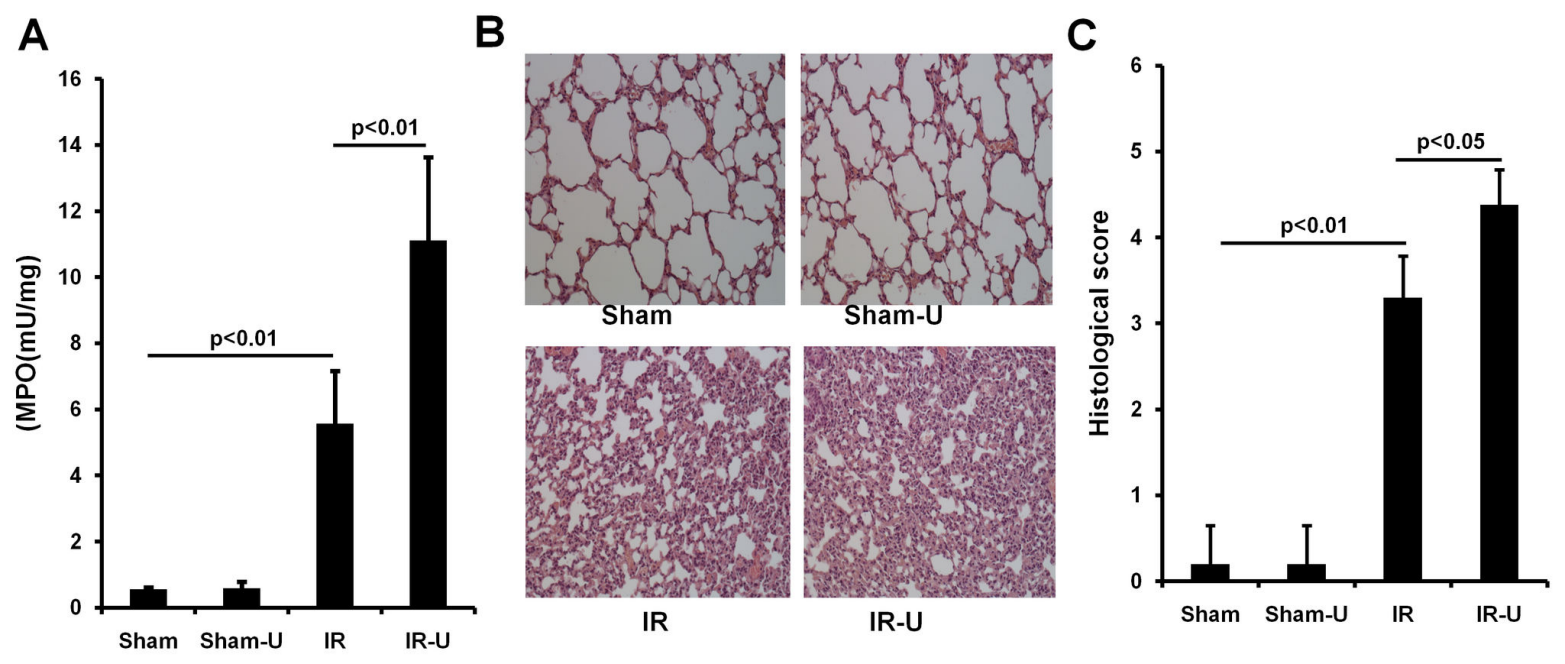
Inhibition of ERK1/2 by U0126 increased lung inflammation and injury after intestinal ischemia/reperfusion. Mice were pretreated with U0126 or vehicle and then subjected to one hour ischemia followed by 6 hours reperfusion in the intestine (n=5). The lung tissue was examined. A. MPO activity by a commercial kit. B. Representative images by HE staining. C. Quantitative tissue damage. Sham-U=Sham-U0126, IR=I/R, IR-U=I/R-U0126.

Intestinal I/R caused significant lung inflammation (sham vs. IR: 0.56±0.07 vs. 5.57±1.59. p<0.01) and injury (sham vs. IR: 0.20±0.45 vs. 3.30±0.48. p<0.01). U0126 further exacerbated both inflammation (IR vs. IR-U0126: 5.57±1.59 vs. 11.11±2.53. p<0.01) and injury (IR vs. IR-U0126: 3.30±0.48 vs. 4.38±0.41. p<0.05). There were no differences in inflammation (sham vs. sham-U0126: 0.56±0.07 vs. 0.59±0.20. p>0.05) or injury (sham vs. sham-U0126: 0.20±0.45 vs. 0.20±0.45. p>0.05) between sham groups (Figure 6).

### Inhibition of ERK1/2 decreased survival after intestinal I/R in vivo

**Figure 7:**
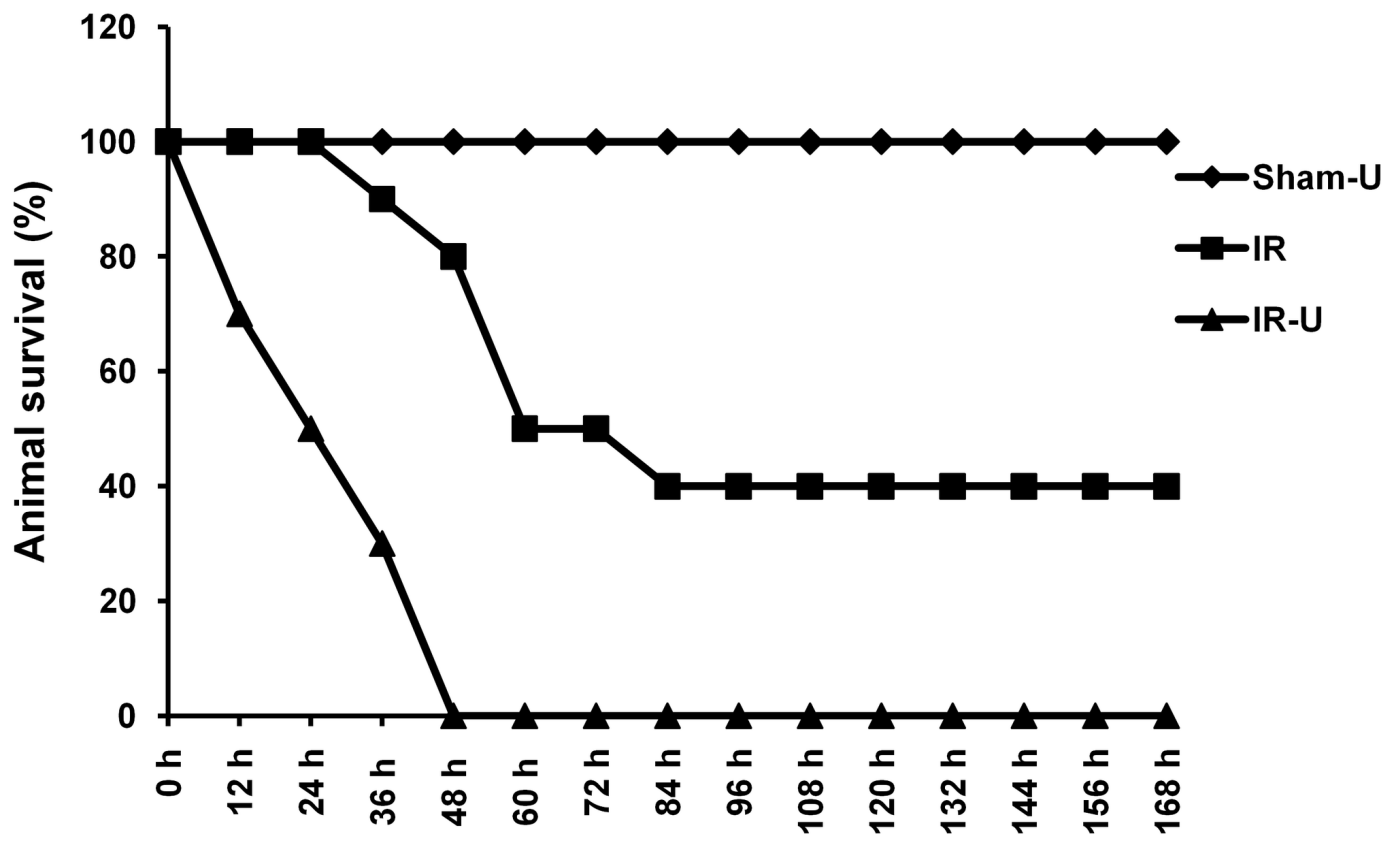
Inhibition of ERK1/2 by U0126 increased animal mortality induced by intestinal ischemia/reperfusion. Mice were pretreated with U0126 or vehicle and then subjected to 75 minutes ischemia and the incision was closed. Animals were observed for 7 days for survival rates (n=10) (IR vs. IR-U0126, p<0.01). Sham-U=Sham-U0126, IR=I/R, IR-U=I/R-U0126.

Since inhibition of ERK1/2 activity by U0126 led to more severe local and remote organ (lung) damage, we assessed the impact of U0126 on mortality and demonstrated a marked increase in mortality in U0126-treated animals (p<0.01) (Figure 7). Mortality was 100% in U0126 treated animals by day four while 40% of vehicle treated animals (4/10) were alive at day seven.

### Inhibition of ERK1/2 decreased p70S6 kinase (p70S6K) activity

Similar to ERK pathway, the mTOR/p70SK pathway is also a major regulator of cell growth, proliferation and motility [22-24] as well as immune responses [25]. ERK1/2 has been shown to regulate the mTOR/p70S6K pathway [26,27]. We therefore examined whether inhibition of ERK1/2 activity also suppressed the activity of p70S6K under our experimental conditions. We found that inhibition of ERK1/2 by U0126 inhibited p70S6K phosphorylation in both H/R exposed intestinal epithelial cells (Figure 1A) and in the reperfused ischemic intestine (Figure 1C). To further confirm the inhibition of ERK1/2 in p70S6K activity, cells were transfected with a p70S6K expressing plasmid (Figure 1B). We found that inhibition of ERK1/2 by U0126 also suppressed the increased activity of p70S6K by p70S6K plasmid transfection (Figure 1B). More importantly, inhibition of ERK1/2 also attenuated the effects of p70S6K overexpression on cell proliferation (empty plasmid+vehicle vs. p70S6K plasmid + vehicle: 2.27±0.31 vs. 3.93±1.01, p<0.01; p70S6K plasmid+vehicle vs. p70S6K plasmid+U0126: 3.93±1.01 vs. 0.97±0.29, p<0.01), migration (empty plasmid+vehicle vs. p70S6K plasmid + vehicle: 91.33±11.50 vs. 152.67±33.50, p<0.01; p70S6K plasmid+vehicle vs. p70S6K plasmid+U0126: 152.67±33.50 vs. 60.33±14.01, p<0.01) and apoptosis (empty plasmid+vehicle vs. p70S6K plasmid + vehicle: 7.03±0.80 vs. 2.50±1.14, p<0.01; p70S6K plasmid+vehicle vs. p70S6K plasmid+U0126: 2.50±1.14 vs. 10.87±1.79, p<0.01) (Figure 2A, 2B and 2C). These suggest that ERK1/2 regulates cell proliferation, migration and apoptosis, at least in part through the, mTOR/p70S6K pathway under intestinal I/R conditions.

## Discussion

U0126 is a signal transduction inhibitor that specifically targets the ERK pathway in mammalian cells [28]. U0126 has been used in vivo to evaluate the effect of ERK1/2 inhibition in a variety of disease models [29,30]. In the present study, we investigated the effect of inhibition of ERK1/2 by U0126 in intestinal I/R injury both in vitro and in vivo. In vitro we found that ERK1/2 inhibition reduced intestinal cell proliferation and migration and promoted apoptosis. In vivo, inhibition increased intestinal inflammation, permeability and injury. It also enhanced systemic inflammation, remote organ (lung) injury and inflammation and importantly, significantly worsened mortality.

ERK1/2 activation controls cell proliferation and apoptosis [7]. Studies have shown the myocardial protection and neuroprotection of activated ERK1/2 [8,31-33]. Recently, Deng et al. reported that activation of ERK1/2 contributed to the protective effect of leptin in intestinal I/R injury. The authors showed that leptin ameliorated intestinal damage coincident with an increase of ERK1/2 activity. Additionally, the ERK1/2 inhibitor, U0126, diminished this benefit [12]. The current study provides further insight into how ERK1/2 protects the intestine. ERK1/2 may enhance intestinal epithelial cell proliferation, reduce apoptosis, and promote cell migration, all crucial to recovery from intestinal damage.

Inhibition of ERK1/2 may also increase inflammation. Our results showed that inhibition of ERK1/2 facilitated neutrophil infiltration into the intestinal tissue and promoted the production of proinflammatory factors systemically during intestinal I/R. Furthermore, ERK1/2 inhibition exacerbated inflammation-related remote organ damage. Consistent with our results, Noh et al. reported that upregulation of ERK1/2 kinase activity promoted the anti-inflammatory cytokine IL-10 expression in lipopolysaccharide-stimulated conditions [34].

The lung is frequently injured after intestinal I/R. Once activated by proinflammatory mediators, neutrophils are able to penetrate the endothelial layer and migrate into the alveolar space [35,36]. Recruitment of neutrophils to the lung is believed to play a critical role in the progression of acute lung injury [37]. We found that suppression of ERK1/2 activity promoted neutrophil accumulation in the lung and increased lung damage.

The precise molecular mechanism of ERK1/2 in regulation of intestinal injury and inflammation after I/R remains to be elucidated. PPARγ is an anti-inflammatory mediator in the intestine and contains a mitogen-activated protein kinase site. Phosphorylation of this site by ERK1/2 resulted in inhibition of PPARγ [38]. However, we have previously shown that PPARγ was activated and protective to the post ischemic gut [39] and in the current study we demonstrated that ERK1/2 was activated under similar conditions. Therefore, our data suggests that it is unlikely that there is a negative relationship between ERK1/2 and PPARγ and that the effect of ERK1/2 on intestinal injury and inflammation is independent of PPARγ.

It is well-known that ERK1/2 regulates mTOR /p70S6K pathway [26,27]. We have demonstrated the detrimental effect of p70S6K inhibition in the intestine after I/R injury. Inhibition of p70S6K by the mTOR inhibitor, rapamycin, aggravated local and systemic inflammation and tissue damage and increased mortality after intestinal I/R [14]. Thus, we explored the possibility that p70S6K mediates the effect of ERK1/2 inhibition on intestinal I/R injury. We demonstrated that in vivo inhibition of ERK1/2 by U0126 markedly reduced intestinal cell proliferation and increased intestinal cell apoptosis along with decreased p70S6K activity in the intestine. In vitro, prevention of ERK1/2 activation by U0126 abolished the activation of p70S6K and attenuated the effect of p70S6K on cell proliferation, apoptosis and migration. Therefore, it is possible that the detrimental effects of ERK1/2 inhibition are in part due to downregulation of p70S6K activity.

In conclusion, we have demonstrated that ERK1/2 activation is crucial to protection against intestinal I/R injury. Inhibition of ERK1/2 decreased cell proliferation, suppressed cell migration, and increased cell apoptosis, as well as exacerbating the breakdown of the intestinal barrier. These effects were due, at least in part, to increased local and systemic inflammation. Additionally, p70S6K mediated the effects of ERK1/2 inhibition on intestinal I/R injury. This finding enhances the understanding of the mechanisms responsible for intestinal I/R injury and may potentially be translated into new therapeutic approaches for prevention and recovery of intestinal I/R damage. The results of this study may also be applicable to other organs damaged by I/R since ERK1/2 is universally expressed by most tissues/organs, such as lung, kidney, liver and heart.
